# The Regularities in Insufficient Leisure-Time Physical Activity in Poland

**DOI:** 10.3390/ijerph13080798

**Published:** 2016-08-08

**Authors:** Elżbieta Biernat, Sonia Buchholtz

**Affiliations:** 1 Collegium of World Economy, Warsaw School of Economics, al. Niepodległości 162, Warszawa 02-554, Poland; 2 Collegium of Economic Analysis, Warsaw School of Economics, al. Niepodległości 162, Warszawa 02-554, Poland; sb43146@sgh.waw.pl

**Keywords:** epidemiology, leisure-time physical activity, demographic and socioeconomic variables, econometric modeling

## Abstract

*Background:* Insufficient physical activity (PA) has become an increasing risk factor of noncommunicable diseases and an important cause of deaths all over the world. The goal of this paper is to provide an in-depth description of insufficient PA in Poland as well as an examination of some of its correlates. *Methods:* We take advantage of statistical and econometric (logistic regression) analysis on the basis of a representative survey. Out of 3056 respondents, we analyze the 1260 low-PA ones. *Results:* The household size is more significant than the household life phase, and only several professions increase the odds of insufficient PA. The influence of socioeconomic status and place of residence is most robust. Gender does not significantly influence insufficient PA. Physical inactivity is concentrated among inhabitants of rural areas and town dwellers, with poor educational profile, and limited labor market opportunities. However, even high socioeconomic status does not completely prevent insufficient activity. *Conclusions:* Groups at the highest risk of inactivity should be covered by promotional actions first. Their aim should mainly be raising the leisure-time physical activity (LTPA) awareness. To start with, primary forms of activity would be walking, Nordic walking and jogging.

## 1. Introduction

Since the 1950s insufficient physical activity (PA) has been recognized as a risk factor of noncommunicable diseases (NCDs) [1], and an important cause of deaths all over the world [2]. Its consequences are already common in developed and developing societies [3]. As the adverse trend has become a hot issue, public health strategies all over the world set the objective of increasing PA among the inactive [4]. There are several recommendations of pro-health PA levels [5], as well as types, exercises and frequencies eligible in prophylactics and treating chronic diseases [6,7].

According to the World Health Organization (WHO) data [7], almost one in four (23.3%) adults in the world has insufficient PA. Its prevalence is not evenly distributed across the globe: in the Americas this share rises to 32.4%, while in Southeast Asia it amounts to 14.7% of the population. Europe ranks slightly above the average (24.5%). These results seem to be underestimating the scale of the phenomenon—as reported by the Center for Disease Control and Prevention and the American College of Sports Medicine (CDC/ACSM), the share of insufficiently activity among adult US citizens exceeds 60% [8] (WHO: 32.4%). Eurobarometer estimates the share of Europe citizens who either do not exercise or do it sparsely at 59% [9].

Increasing physical activity, particularly leisure-time physical activity (LTPA), is then a priority task for public health policy-makers worldwide. It has been known that leisure-time effort in the form of training with the duration and frequency recommended for maintaining health may, regardless of the intensity of occupational PA, raise individual capacity and physical fitness [10]. It may also bring significant health benefits, including reduced risk of death due to chronic diseases [11]. In contrast with occupational PA, in LTPA the dynamic body movements vary, and intensive efforts lasting for short periods take place in intervals, interchangeably with active or passive rest [12]. In fact, for many occupations LTPA has become the only chance to counteract sedentary behavior patterns [13].

Unfortunately, 52% of Poles are physically inactive [9] and their passive attitude towards physical effort coincides with ruthless epidemiological statistics—cardiovascular diseases are responsible for 46% of deaths, malignancies for a further 24.5% [14]. At the same time, overweight characterizes 36.4% of Poles and another 15.8% are obese [15]. Moreover, 54% struggle with hypertension, while 22% of adult Poles consult a doctor due to musculoskeletal diseases [16]. Piekutowska et al. partially attribute this state of things to the inefficiency of the previous actions aimed at promoting LTPA and preventing obesity in Poland [17]. However, designing an adequate policy mix requires good recognition of PA across various social sections [18]. 

The objective of this paper is to provide an in-depth description of insufficient PA in Poland, as well as an examination of some of its correlates. By employing econometric modeling, it will support the public health policy, for instance in designing targeted pro-health campaigns as well as measures aimed at reducing the inequality in health status. 

## 2. Materials and Methods 

### 2.1. Dataset

The survey was commissioned by the Ministry of Sport and Tourism of the Republic of Poland and conducted by GfK Polonia [19]. It is an International Physical Activity Questionnaire (IPAQ) compatible, computer-assisted personal interview run every six months since early 2014. A representative sample of adult Poles (aged 15–69) are asked about their physical activity (type, frequency, intensity), as well as various demographic and socioeconomic characteristics. The data are available for three out of four waves of the interviews. 

Each wave examined approximately 1000 respondents, although this is a bit insufficient to draw robust conclusions regarding several cross-sections of the data. However, as we analyze cross-regional datasets measured in close intervals, questionnaires do not differ significantly and activity distributions among the first seven deciles of respondents across waves are similar, so we decided to pool the data from consecutive waves. The pooled database consists of 3056 records, out of which 1260 respondents were described as low physical activity (0–600 MET-min/week during seven days prior to the day of survey): 715 with literally no activity (0 MET-min/week) and 545 with a positive but insufficient total amount of activity (1–600 MET-min/week). According to the IPAQ guidelines, only activity lasting at least 10 min without interruption was recorded. Moreover, cases of unreasonably high total physical effort (over 16 h) should be excluded from the analysis. Similarly, for the respondents who declared daily effort of one type of over 3 h records are automatically corrected to 3 h. As a result, 11 unreliable observations were dropped. The final subsample contains 1260 low physical activity respondents, who are the subject of our analysis (Table 1). 

IPAQ-compatibility of the Polish survey allows for international comparisons of the results all over the world. We use its long version [20]. The questionnaire distinguishes between LTPA (including: vigorous activity (VPA), moderate activity (MPA), and recreational walking) and transportation-related PA (walking or cycling from one place to another). We decided to include types of PA that contribute to meeting the WHO recommendations and are a result of respondents’ voluntary decisions. The descriptive statistics of physical effort are converted into average energy expenditure of each activity in MET (MET unit corresponds to O_2_ consumption during rest and equals to 3.5 mL O_2_/kg of the body mass per minute), according to a formula (each minute of activity equals to: VPA—8.0 MET, MPA—4.0 MET, walking—3.3 MET, cycling—6.0 MET).

By multiplying the frequency of activity per week and the effort, we obtain the sum of energy expenditures for each activity. After summarizing over all types of PA, we are able to assign the respondents into low, moderate or high physically active categories. Low physically activity: 0–600 MET-min/week, moderate: 601–1500 MET-min/week, high: 1501 and more MET-min/week. Despite controversies [21], these thresholds refer to all age groups, as in the original IPAQ questionnaire [22,23]. The low physical activity group is in the limelight of the analysis. For further analysis we find it useful to distinguish between individuals completely inactive and those whose PA is insufficient to meet the WHO guidelines [22]. Henceforth, we use notions of inactive for individuals with 0 MET-min/week of PA and insufficiently active for those with 1–600 MET-min/week of PA. These two categories contrasted with the sufficiently active group, with over 600 MET-min/week. 

### 2.2. Methods

Being fully aware of the limitations arising from self-assessment of physical activity [21,22], we constructed a logistic regression. Its binary dependent variable (low physical activity) denotes 1 for respondents whose physical effort per week does not exceed 600 MET-min and 0 otherwise, so as to examine demographic and socioeconomic variables correlated with insufficient activity. The original set of explanatory variables covers: gender (male, female), age (15–29, 30–39, 40–49, 50–59, 60–69), household size (1, 2, 3, 4, 5 and more members), household life phase (eight categories: students cohabiting with parents, workers cohabiting with parents, young non-cohabiting households, families with youngest child aged: 0–6/7–14/15–25, senior working/retired households), professional status (executives or owners, high-level white-collar workers, low-level white-collar workers, qualified blue-collar worker, non-qualified blue-collar worker, farmer, unemployed, homemaker, pupil/student, pensioner or old-age pensioner), socioeconomic status (SES; upper middle class, middle class, lower middle class and skilled working class, working class, non-working class), educational level (higher, secondary, vocational, primary education), Internet use (frequent, rare, never), place of residence (urban areas with over 500 K inhabitants, urban areas with 50 K–500 K inhabitants, urban areas with less than 50 K inhabitants, rural areas) and country region (five macroregions of Poland). Except for the profession and SES, all the questions were asked directly. Profession is classified by the survey company into one of the abovementioned categories on the basis of undisclosed questions. Similarly, SES is an artificial variable representing joint income, educational level and profession. In the descriptive analysis we include all the available characteristics while their potential overlapping is tested in the statistical analysis section. 

As we use a representative micro-database (for each respondent we have a detailed set of characteristics and an analytical weight reflecting how strong the representation of the respondent’s characteristics in the society is), additional statistical calculations supplement the econometric modeling. While the former presents the relative frequency of physical inactivity and insufficient physical activity among Polish adults, the latter analyzes correlations between a wide range of demographic and socioeconomic characteristics, and insufficient physical activity. The scientific aim notwithstanding, such analysis is the first stage in designing effective policy towards increasing of physical activity. Statistical calculations were carried out with SPSS^®^ Statistics Version 21 software (IBM^®^, Armonk, NY, USA) econometric modeling—with STATA 14 software (StataCorp LP, Lakeway Drive, TX, USA). 

## 3. Results

### 3.1. Descriptive Analysis

The survey reveals the scale of physical inactivity in Poland: almost one in four adults declares complete inactivity, while over two in five make too little physical effort to meet the WHO guidelines (Table 2). The distribution of PA effort among the insufficiently active is roughly even—the mean effort for this group equals 350 MET-min/week (SD = 156). Cross-sectional analysis of non-active and insufficiently active respondents delivers robust results—except for gender, the *p*-value remains below the level of 0.01. 

**Figure 1:**
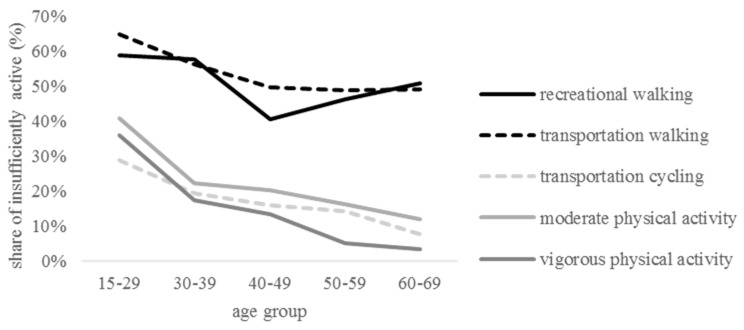
Share of insufficiently active taking up physical activity, by age groups and type of physical activity.

As individuals age and the share of inactivity increases, so does the share of low physical activity. The respective proportions double between the youngest and the oldest age group. Out of all types of effort, walking (regardless of aim) is the most popular one among the insufficiently active, and does not lose its popularity among older groups. The share of transportation walkers saturates at the level of 50%, while recreational walking regains popularity among those who are 50+. By contrast, commutative cycling, MPA and VPA decrease with age (Figure 1). 

The share of the non-active is low among individuals living with parents and much lower than among non-senior childless households. As families expand and children grow, parents gradually reduce their PA. The shares for parents of adult children are similar to senior households. Recreational walking happens more often among students cohabiting with parents, non-senior childless households, as well as parents of the youngest children. Also MPA is more frequent among households with children (12%–21%). VPA gains popularity in non-senior childless households (5%), and so does cycling (9%). Household size, even though significant, does not exhibit a clear pattern of physical inactivity.

White-collar workers are in general more active than blue-collar workers, with the exception of non-qualified blue-collar workers, whose activity pattern is similar to high-level professionals. An extreme case of inactivity is found among farmers. Physical and economic inactivity go together: the unemployed, homemakers and pensioners exhibit too little physical effort more often than their working counterparts.

In general, the higher the level of education, the higher the probability of fulfilling the WHO recommendations. Walking is more frequent among people with primary education (72%, comparing to ca. 50% in other groups), cycling is a domain of secondary education holders (6%, comparing to 0% in tertiary, 3% in primary and 4% in vocational), while interest in MPA increases with educational level (from 7% among primary to 18% among tertiary education holders). 

Activity is a monotonically increasing function of socioeconomic status. Even though the overall activity pattern is clear, there is none when it comes to the types of activity. The only exception in this case is MPA, significantly more frequent among the middle class (17%), and cycling, characteristic for the lower middle class and skilled working class (8%).

Low PA is much more frequently observed among rural than urban dwellers. One in three does not engage in any activity, while among the inhabitants of Poland’s five biggest cities (over 500 K inhabitants) it happens in one in 10 cases. The smaller the place of residence, the more common the occurrence of PA deficits. Lack of recreational walking was typical of rural dwellers (63%; it is a slightly more popular form of PA among urban dwellers: 52%–57%), while the reverse applies to walking for the purpose of transportation (48% vs. 56%–61%). MPA is taken up more often in bigger cities (18%) and in rural areas (14%) than in towns (11%–12%), while cycling is most common in rural areas (9%) and smaller towns (5%). In cities, the share of cyclists in negligible. Neither the region of residence itself nor its cross-section with the place of residence show any patterns. 

### 3.2. Statistical Analysis

The descriptive analysis confirms several intuitions on regularities among those with low physical activity in Poland. However, it is unable to identify which factors affect their low activity. In the next step we examine whether the existing concentration of insufficient activity is statistically significant. In order to answer the question, we use logistic regression (*n* = 3028). The Hosmer-Lemeshow test indicates that the model is well fitted (Table 3). 

Contrary to the existing literature, gender does not significantly influence insufficient physical activity in Poland. Only partial results are obtained by contrasting age groups. Compared to the youngest age group (15–29), the middle-agers are more prone to low PA by 70% (30–39) and 80% (40–49). A similar proportion holds for individuals in their 50s, 60s and older; however, the significance is only slightly above the 0.05 threshold. Adding interactions of gender and age group fails to deliver any added value in uncovering patterns of physical inactivity (lack of statistical significance). 

The size of the household is more significant than the household life phase. When compared with one-person households, each marginal member increases the odds of insufficient activity—the second one by 51%, the third by 67%. For four and more members, however, statistical significance of the effect decreases. Among household types, the only significant one was the status of a student cohabiting with his or her parents. In this group the odds of insufficient activity decreased by over 72% when confronted with a non-senior, economically active childless household. The non-significance of various family categories is robust, regardless of its character. 

Similarly, only several professions are significantly correlated with low physical activity. The strongest relation applies to farmers whose odds of insufficient activity—compared to white-collar workers—are almost 2.5 times higher. For professionals this ratio is 2.2. Also, the position of an executive, manager or owner and a skilled blue-collar worker affects the low PA to some extent—the respective ratios are 1.9 and 1.6 times (compared to a white-collar worker). Among the economically inactive, the only significant category is the pensioners with an odds ratio of almost 1.8 compared to the reference group.

According to the model, not the demographic variables, but socioeconomic variables are of extreme importance when it comes to identifying the correlates of insufficient physical activity in Poland. The lower the socioeconomic status, the higher the odds are of insufficient activity and this seems to be a very robust conclusion. Compared to the upper middle class, the lower middle class and skilled working class are characterized by over 80% higher odds of insufficient activity. For working class members this proportion exceeds 110%, while for the non-working it is almost three times as high. Similarly, strong conclusions arise if the place of residence is included as a regressor. Rural area dwellers and town dwellers have visibly higher odds of insufficient physical activity than inhabitants of big cities—by over 110% and 60%, respectively. 

## 4. Discussion

The general purpose of this paper was to characterize insufficient physical activity among Polish adults and identify its correlates. This seems to be an urgent issue as more than 40% of citizens make smaller physical efforts than the level that would keep them healthy, according to the WHO standards [7]. Over half of those with low physical activity declare literally no activity, either LTPA or transportation-induced. Besides, 25% of adult Poles are non-walkers, which is one of the highest proportions among EU member states [9]. In this light, a high risk of NCDs is justified. In a country of over 37 million inhabitants, these shares translate into millions of people at risk of NCDs. 

Although the increase in inactivity in Poland (3.3 percentage points over the period of 2009–2013) is a part of an international trend [9], there are several factors suggesting that the trend of physical inactivity will strengthen, unless the public policy becomes more successful. Firstly, provided the age-specific patterns of activity remain stable, the population aging in Poland [24] should, on average, reduce activity among individuals. Until now senior citizens have not been convinced to be physically active, even though they retire relatively early [25] and are at low risk of poverty [26]. As indicated by European Agency for Safety and Health at Work (EU-OSHA), PA should become an element of the actions aimed at preventing early retirement and increasing employability [27], including developing a health prevention profile of an employee with standard tests for PA measurement or annual PA level tests for senior citizens recorded in the national health monitoring system. Moreover, keeping in mind their complaints on subjective health status [28], considering regular, moderate PA could—at least to some extent—solve their problems [29,30]. 

Secondly, the adverse trend will be influenced by the decreasing activity of the youngest age groups. Existing literature suggests that physical inactivity among today’s children is a massive problem [31,32], mainly because a sedentary lifestyle and avoidance of compulsory physical education classes result in poor behavioral patterns in later phases of life [33]. The first signals of inactivity are observed in the dispersion in our subsample: while the overall performance of the youngest age group is relatively good, we identify a share of those avoiding any PA, even walking. If these patterns are at least partially transferable between generations—as confirmed by other researchers [34,35]—the intervention should not be limited to children only.

Thirdly, physical activity in Poland is frequently performed as long as various duties allow for it, but additional professional or family commitments reduce physical effort. Moreover, even when commitments are reduced in scale, low PA levels persist. This conclusion is confirmed by the Polish Public Opinion Research Center survey [36]: Poles do not perceive physical activity as a desired form of free-time activity. Sedentary rest dominates, going for a walk is undertaken occasionally (24%), and a physical workout is even less popular (11%). Even though Poles complain about their lack of free time, they would spend it neither on sport (7%), nor going for a walk or cycling (4%). 

Another issue worth mentioning is the notable variation in physical effort between cross-sectional subsamples. The most physically active subsections consist of less than 20% of low physically active, while for the least active subsections the respective share exceeds 60%, posing a threat to social cohesion in this field. In many cases, the decision about physical activity is affected by financial availability. This argument seems to be well justified by the preference of transportation walking among the economically active and students, as well as cycling among rural area dwellers. In our research, the latter are characterized by lower PA and the logistic regression confirms the robustness of this relationship. This conclusion is in line with previous research indicating that, among rural area dwellers, the probability of meeting the recommended PA amount is significantly lower than among urban dwellers [37,38]. Moreover, city dwellers show a much higher interest in MPA than rural area dwellers (often working in urban areas), and especially town dwellers. These decisions are probably shaped by transaction costs, i.e., financial and time availability of various sport infrastructures, namely football fields, fitness clubs, cycle paths, playgrounds or simply parks and forests. Those are unevenly distributed in Poland [39]. As Parks, Castro, and King prove [40], being too far from activity facilities, lower social support, and less pavements are usually major barriers for living actively in rural areas. Unfortunately, it would also confirm that physical activity is affected by social inequalities. 

However, financial constraints do not justify the walking patterns. Sport is generally perceived in Poland as an issue separate from health and not as its significant element. This lack of awareness is especially visible when physical inactivity among the poorly educated and those with financial constraints is analyzed. The better educated are more prone to follow the rules of a healthy lifestyle, take care of their health and control it regularly, exercise, and generally spend time actively. They are also more educated as to threats to physical and psychological well-being, and are able to avoid negative consequences of these states and minimize their adverse effects [41]. The existing literature suggests that, to some extent, individuals change their unhealthy habits when they are made aware of their consequences [42]. This observation finds confirmation in our dataset. Among adults with primary or vocational education, the share of inactivity is twice as high compared with people holding higher or secondary degrees. Moreover, white-collar workers are more active than blue-collar workers (in the details it is not as clear-cut [43,44]) and especially farmers, in whose case technological changes in their work conditions have taken place, but their physical activity has not been adjusted yet [45,46]. Economic inactivity (except for students) and the resulting free time are not reflected in their PAs. This case is ambiguous as the unemployed have obvious difficulties in fulfilling their pro-health aims (unable to purchase pro-health goods and services). However, it is not an obstacle when it comes to either leisure or transportation walking. This finding is confirmed also by Pampel, Krueger, and Denney [47]. In this context, the socioeconomic status, which has been strongly confirmed in the modeling procedure, is even more distinctive. Higher education, high professional status and related incomes go together with the share of the sufficiently active and the duration of LTPA [48,49]. High SES is associated with caring for one’s own health, while a professional career demands good fitness, stress resilience, and a slim figure [50,51]. By contrast, low SES considerably decreases the awareness of health issues and motivation, as well as imposes financial constraints [52]. Empirically, among individuals with lower occupational status, LTPA is crowded out by physical activity at work, during household chores and in commuting [53,54]. However, it is worth emphasizing that neither education nor profession or status provide enough physical effort. 

This study sheds a new light on physical inactivity in Poland. However, there are several limitations that reduce the robustness of our conclusions. Firstly, what would increase our knowledge in physical activity patterns is a panel dataset. It would show the actual changes in physical activity when a life event occurs. It would also allow us to distinguish between real changes and cohort effects. The crucial issue in this context is whether individuals reduce their effort as time goes by or if they are time-consistent. Each pattern requires slightly different means. Secondly, the sample of 1000 respondents has limited robustness when drawing conclusions. Thirdly, declaration and self-assessment are prone to exaggeration, which we assume to be negligible. This factor could be irrelevant if objective measurements replaced the declarations. 

## 5. Conclusions

Increasing physical activity, particularly LTPA, is a priority task for public health policy worldwide. In Poland, there should be special attention paid to it because as many as 40% of its citizens (analyzed in terms of LTPA and transportation-related PA) do not meet the WHO health standards (including more than 50% declaring literally no activity). In a country of over 37 million inhabitants, these shares translate into millions of people at risk of NCDs. As the consequences of complete inactivity are substantial both in terms of financial costs and untapped potential, the preventive actions should be primarily aimed at sport-averse individuals. Insufficient activity is most frequently observed among inhabitants of rural areas and town dwellers, those with a poor educational profile, and those limited labor market opportunities (economically inactive, farmers). These groups should be covered by promotional actions first. However, even high socioeconomic status (e.g., managers and professionals) does not prevent insufficient activity completely.

PA is rarely perceived as an attractive leisure-time activity; therefore, educational programs should be aimed at raising the awareness of LTPA. For groups at risk of inactivity with limited knowledge and financial constraints, basic forms of activity should be promoted, and that includes walking, Nordic walking or jogging. These disciplines seem to be effective also in the case of elder individuals. 

## Figures and Tables

**Table 1:** The sample structure.

Age Group	Male Wave 1	Male Wave 2	Male Wave 3	Male Total	Female Wave 1	Female Wave 2	Female Wave 3	Female Total
15–29	29	40	29	358	24	19	28	329
30–39	48	45	37	295	36	29	40	290
40–49	40	25	37	229	30	27	30	226
50–59	43	44	45	245	42	39	40	265
60–69	42	55	63	353	82	86	86	466
Total	202	209	211	622	214	200	224	638

**Table 2:** Structure of physical inactivity and low activity in Poland (%).

Variables (*p*-Value)		Inactive (0 MET-min/week) *n* = 715	Insufficiently Active (1–600 MET-min/week) *n* = 545	Low Physically Active (0–600 MET-min/week) *n* = 1260
	total	24.0	17.1	41.1
Gender *p* = 0.225	female	23.6	17.3	40.9
	male	24.5	16.9	41.3
Age group *p* = 0.005	15–29	14.1	10.5	24.6
	30–39	21.5	18.4	39.8
	40–49	30.3	15.1	45.4
	50–59	29.0	18.9	47.9
	60–69	28.4	22.5	50.9
Household size *p* = 0.001	1	23.3	19.6	42.8
	2	25.7	22.1	47.8
	3	23.4	16.2	39.6
	4	20.6	15.9	36.5
	5 and over	26.4	13.8	40.2
Household life phase *p* = 0.000	studying, living with parents	11.5	6.5	18.0
	working, living with parents	16.0	14.6	30.5
	non-senior, working, childless, separate living	18.9	14.4	33.3
	household with children 0–6	20.0	15.5	35.5
	household with children 7–14	25.7	16.4	42.1
	household with children 15–25	31.0	19.3	50.3
	senior, working	28.8	19.3	48.1
	senior, not working	27.6	23.0	50.6
Education *p* = 0.000	primary	43.0	19.6	62.5
	vocational	31.6	19.9	51.5
	secondary	19.1	19.3	38.4
	higher	15.4	17.2	32.6
Use of Internet *p* = 0.000	everyday	14.0	15.0	29.0
	rare	26.3	16.8	43.1
	never	34.1	19.7	53.9
Profession *p* = 0.000	managers, executives, owners	18.0	15.8	33.9
	high-level professionals	20.6	17.0	37.6
	low-level professionals	15.0	9.4	24.4
	qualified blue-collar jobs	23.2	17.8	40.9
	non-qualified blue-collar jobs	20.7	16.8	37.5
	farmers	44.5	21.2	65.6
	homemakers	25.1	20.5	45.6
	students	13.1	6.3	19.3
	pensioners	27.6	21.8	49.4
	unemployed	23.8	14.8	38.5
Socioeconomic status *p* = 0.000	upper middle class	14.3	14.4	28.6
	middle class	16.7	16.9	33.7
	lower middle class and skilled working class	23.9	16.9	40.8
	working class	29.6	17.6	47.2
	not working	36.2	19.5	55.7
Place of residence *p* = 0.000	rural areas	31.7	17.5	49.3
	urban areas, less than 50 K inhabitants	25.1	16.9	42.0
	urban areas, 50 K–500 K inhabitants	17.2	16.3	33.5
	urban areas, over 500 K inhabitants	10.6	17.7	28.3
Region *p* = 0.003	central	18.6	20.3	38.9
	north-eastern	29.4	18.5	48.0
	north-western	21.3	16.5	37.8
	south-western	26.0	14.2	40.2
	south-eastern	27.3	17.4	44.7

Shares in category education are calculated for age groups 30 and over. *p*-Value reflects a significance of difference across categories for a given factor.

**Table 3:** Logit estimation of physical insufficient activity in Poland.

Variables		Odds Ratio	Standard Error	*p*-Value	95% Confidence Intervals
	constant	0.0835	0.0299	0.000	0.0414–0.1684
Gender	female	ref.			
	male	0.9327	0.0982	0.509	0.7587–1.1467
Age group	15–29	ref.			
	30–39	1.6974	0.3293	0.006	1.1604–2.4830
	40–49	1.8090	0.4323	0.013	1.1322–2.8902
	50–59	1.6261	0.4108	0.054	0.9909–2.6685
	60 and over	1.7818	0.5267	0.051	0.9980–3.1812
Household size	1	ref.			
	2	1.5114	0.2230	0.005	1.1317–2.0185
	3	1.6651	0.3541	0.017	1.0974–2.5267
	4	1.5390	0.3724	0.075	0.9576–2.4734
	5 and over	1.5202	0.3958	0.108	0.9124–2.5328
Household life phase	studying, living with parents	0.2323	0.1733	0.050	0.0538–1.0028
	working, living with parents	0.8003	0.2399	0.458	0.4446–1.4406
	non-senior, working, childless, separate living	ref.			
	household with children 0–6	0.6421	0.1850	0.124	0.3650–1.1295
	household with children 7–14	0.7862	0.2345	0.420	0.4380–1.4111
	household with children 15–25	1.0246	0.3315	0.940	0.5433–1.9323
	senior, working	1.1461	0.3225	0.628	0.6601–1.9900
	senior, not working	0.9397	0.3023	0.847	0.5001–1.7659
Profession	managers, executives, owners	1.9138	0.5911	0.036	1.0445–3.5068
	professionals	2.1602	0.5651	0.003	1.2934–3.6078
	white-collar jobs	ref.			
	qualified blue-collar jobs	1.5972	0.3568	0.036	1.0308–2.4750
	non-qualified blue-collar jobs	1.0459	0.3389	0.890	0.5540–1.9744
	farmers	2.4256	0.7683	0.005	1.3035–4.5139
	homemakers	1.6831	0.5608	0.118	0.8757–3.2349
	students	2.0347	1.4297	0.312	8.0697–8.0697
	pensioners	1.7992	0.5303	0.046	1.0094–3.2068
	unemployed	1.2012	0.3369	0.514	0.6930–2.0820
Socioeconomic status	upper middle class	ref.			
	middle class	1.3087	0.2964	0.235	0.8393–2.0405
	lower middle class and skilled working class	1.8118	0.4162	0.010	1.1548–2.8425
	working class	2.1158	0.5288	0.003	1.2961–3.4538
	not working	3.7999	1.1477	0.000	2.1018–6.8701
Place of residence	rural areas	2.1107	0.3867	0.000	1.4736–3.0231
	urban areas, less than 50 K inhabitants	1.6426	0.3006	0.007	1.1474–2.3515
	urban areas, 50 K–500 K inhabitants	1.1123	0.2023	0.559	0.7787–1.5888
	urban areas, over 500 K inhabitants	ref.			

*n* = 3028; ref. – reference category; McFadden’s *R*-squared: 0.0757; Hosmer-Lemeshow test: 0.5943.

## Abbreviations

The following abbreviations are used in this manuscript:

PA	Physical Activity
NCD	Noncommunicable Disease
WHO	World Health Organizations
LTPA	Leisure-Time Physical Activity
IPAQ	International Physical Activity Questionnaire
VPA	Vigorous Physical Activity
MPA	Moderate Physical Activity
EU-OSHA	European Agency for Safety and Health at Work
